# Association between cyclooxygenase-2 (*COX-2*) 8473 T > C polymorphism and cancer risk: a meta-analysis and trial sequential analysis

**DOI:** 10.1186/s12885-018-4753-3

**Published:** 2018-08-24

**Authors:** Qiuping Li, Chao Ma, Zhihui Zhang, Suhua Chen, Weiguo Zhi, Lei Zhang, Guoyao Zhang, Lei Shi, Fei Cao, Tianjiang Ma

**Affiliations:** Department of Medical Oncology, Luohe Central Hospital, Luohe First People’s Hospital, No. 56 People’s East Road, Luohe City, 462000 Henan Province China

**Keywords:** *COX-2* gene, 8473 T > C polymorphism, Cancer, Risk, Meta-analysis

## Abstract

**Background:**

Numerous studies have investigated the relationship between *COX-2* 8473 T > C polymorphism and cancer susceptibility, however, the results remain controversial. Therefore, we carried out the present meta-analysis to obtain a more accurate assessment of this potential association.

**Methods:**

In this meta-analysis, 79 case-control studies were included with a total of 38,634 cases and 55,206 controls. We searched all relevant articles published in PubMed, EMBASE, OVID, Web of Science, CNKI and Wanfang Data, till September 29, 2017. The pooled odds ratios (ORs) with 95% confidence intervals (CIs) were used to evaluate the strength of the association. We performed subgroup analysis according to ethnicity, source of controls, genotyping method and cancer type. Moreover, Trial sequential analysis (TSA) was implemented to decrease the risk of type I error and estimate whether the current evidence of the results was sufficient and conclusive.

**Results:**

Overall, our results indicated that 8473 T > C polymorphism was not associated with cancer susceptibility. However, stratified analysis showed that the polymorphism was associated with a statistically significant decreased risk for nasopharyngeal cancer and bladder cancer, but an increased risk for esophageal cancer and skin cancer. Interestingly, TSA demonstrated that the evidence of the result was sufficient in this study.

**Conclusion:**

No significant association between *COX-2* 8473 T > C polymorphism and cancer risk was detected.

**Electronic supplementary material:**

The online version of this article (10.1186/s12885-018-4753-3) contains supplementary material, which is available to authorized users.

## Background

Currently, cancer is still considered as a global public health problem and the leading cause of human death [1], with an estimate of 14.1 million new cancer cases and 8.2 million cancer deaths in 2012 worldwide [2]. A large number of epidemiological and biological researches have demonstrated that cancer, as a multifactorial disease, is caused by a series of potential risk factors, including genetic and environmental factors [3]. However, the accurate mechanisms of carcinogenesis remained unclear. In recent years, many studies have pointed that the expression of tumor suppressor genes and oncogenes is closely associated with inflammation, which can also promote the transformation of cancer [4–6].

Cyclooxygenase-2 (COX-2), also called prostaglandin endoperoxide synthetase (PTGS-2), is an inducible isoform of COX enzyme that converts arachidonic acid to prostaglandins, and prostaglandins are generally regarded as the effective mediators of inflammation [7]. By producing prostaglandins, COX-2 is considered to participate in several biological processes, such as carcinogenesis, cell proliferation, angiogenesis and mediating immune suppression. More and more evidence has pointed that increased expression of *COX-2* is closely associated with malignant progression [8–10]. In addition, it is also shown that carcinogenesis could be prevented by using selective COX-2 inhibitors [11]. The human *COX-2* gene, with a length of 8.3 kb and consisting of 10 exons, is located on chromosome lq25.2-q25.3. Different polymorphism sites in the *COX-2* gene have been clarified. One of these functional polymorphisms, the 8473 T > C polymorphism in the 3′-untranslated region (3’UTR) of *COX-2* gene is the most widely investigated polymorphism.

Previous functional researches have indicated that 8473 T > C polymorphism is related to the alteration of the mRNA level of *COX-2* gene via playing an important role in message stability and translational efficiency [12]. There are numerous case-control studies that have investigated the role of 8473 T > C polymorphism in cancer risk. However, the results of these studies remain inconclusive. Therefore, to draw a more precise conclusion, we conduct the present meta-analysis to evaluate the association of 8473 T > C polymorphism in *COX-2* gene with cancer susceptibility.

## Methods

### Identification and eligibility of relevant studies

Literature in electronic databases, including PubMed, EMBASE, OVID and Web of Science, were systematically searched using the following terms: “cyclooxygenase-2 or *COX-2* or *PTGS2*” and “polymorphism or variant or genotype” and “cancer or carcinoma or neoplasm”. To expand our investigation, we also searched China National Knowledge Infrastructure (CNKI) and Wanfang Data using the corresponding Chinese terms. Furthermore, references cited in each included study were also searched manually to identify potential additional relevant studies. When the information provided in the article was unclear, we contacted the author for detailed raw data. If data were overlapping, we adopted the most recent and comprehensive research for this meta-analysis. The last search date was September 29, 2017.

### Inclusion and exclusion criteria

The inclusion criteria were as follows: studies investigating the association of *COX-2* 8473 T > C polymorphism with cancer risk; studies with essential information on genotype or allele frequencies to estimate ORs and 95% CIs; studies with human subjects; and case-controlled studies. Exclusion criteria included: reviews or meta-analyses; animal or cytology experiments; duplicate publications; studies not involving cancer; no controls, not according with Hardy-Weinberg equilibrium (*P*_HWE_ < 0.05) in the control group, and studies published neither in English nor Chinese.

### Data extraction

From all eligible publications, the following data, including the first author, year of publication, population ethnicity, country, source of controls, cancer type, detection genotype methods of *COX-2* 8473 T > C polymorphism, and number of cases and controls, were carefully extracted by two authors (Qiuping Li and Chao Ma) independently. Inconsistencies were resolved after discussion, and a consensus was reached for all extracted data.

### Quality assessment

The quality of the included studies was evaluated using the Newcastle–Ottawa scale (NOS) [13] with eight items (Additional file 1: Table S1). We awarded a study a maximum of nine star scale based on selection (four stars maximum), comparability (two stars maximum) and exposure (three stars maximum). Studies with NOS scores of 1–3, 4–6 and 7–9 were considered as low-quality, medium-quality and high-quality studies, respectively. Medium-quality and high-quality studies were included in the present meta-analysis.

### Statistical analysis

We analyzed the association of *COX-2* 8473 T > C polymorphism with cancer risk using Stata software (Version 11.0; StataCorp, College Station, TX). Cumulative ORs and the corresponding 95% CIs were employed to measure the strength of associations. All *p* values were two-sided, and *p* < 0.05 was considered as statistically significant. Heterogeneity was assessed using a Q statistic (considered significant heterogeneity among the studies if *P* value< 0.10) and an I-squared (I^2^) value [14]. When heterogeneity of studies was significant, the DerSimonian and Laird random-effects model [15] was performed to calculate the pooled ORs. Otherwise, the Mantel–Haenszel fixed-effects model was used [16]. We performed the sensitivity analysis to explore heterogeneity when significant heterogeneity was detected. Subgroup analysis was used to explore the effect of ethnicity, study design, cancer type and genotype method. Moreover, publication bias was evaluated quantitatively using Begg’s [17] and Egger’s [18] tests. Significant publication bias was indicated if *P* value< 0.05.

### Trial sequential analysis

Type I errors may be caused by meta-analysis due to random error because of insufficient sample size in this meta-analysis. And the conclusions of the meta-analysis tended to be changed by later studies with a larger sample size [19]. When TSA was performed in a meta-analysis, both inadequate information size and false positive conclusions were revealed, and the above limitations were also overcome [19, 20]. Therefore, we used TSA software version 0.9 beta in this meta-analysis on the basis of two-sided tests, with an overall type I error risk of 5%, a statistical test power of 80%, and relative risk reduction of 10%. Trails were ignored in interim due to too low information to use (< 1.0%) by the TSA software. When the cumulative Z-curve in results crosses the TSA boundary or enters the insignificance area, a sufficient level of evidence has been reached, and no further studies are necessary. However, when the Z curve does not exceed any of the boundaries and the required sample size has not been reached, evidence to reach a conclusion is insufficient [21].

## Results

### Characteristics of the included studies

**Fig. 1:**
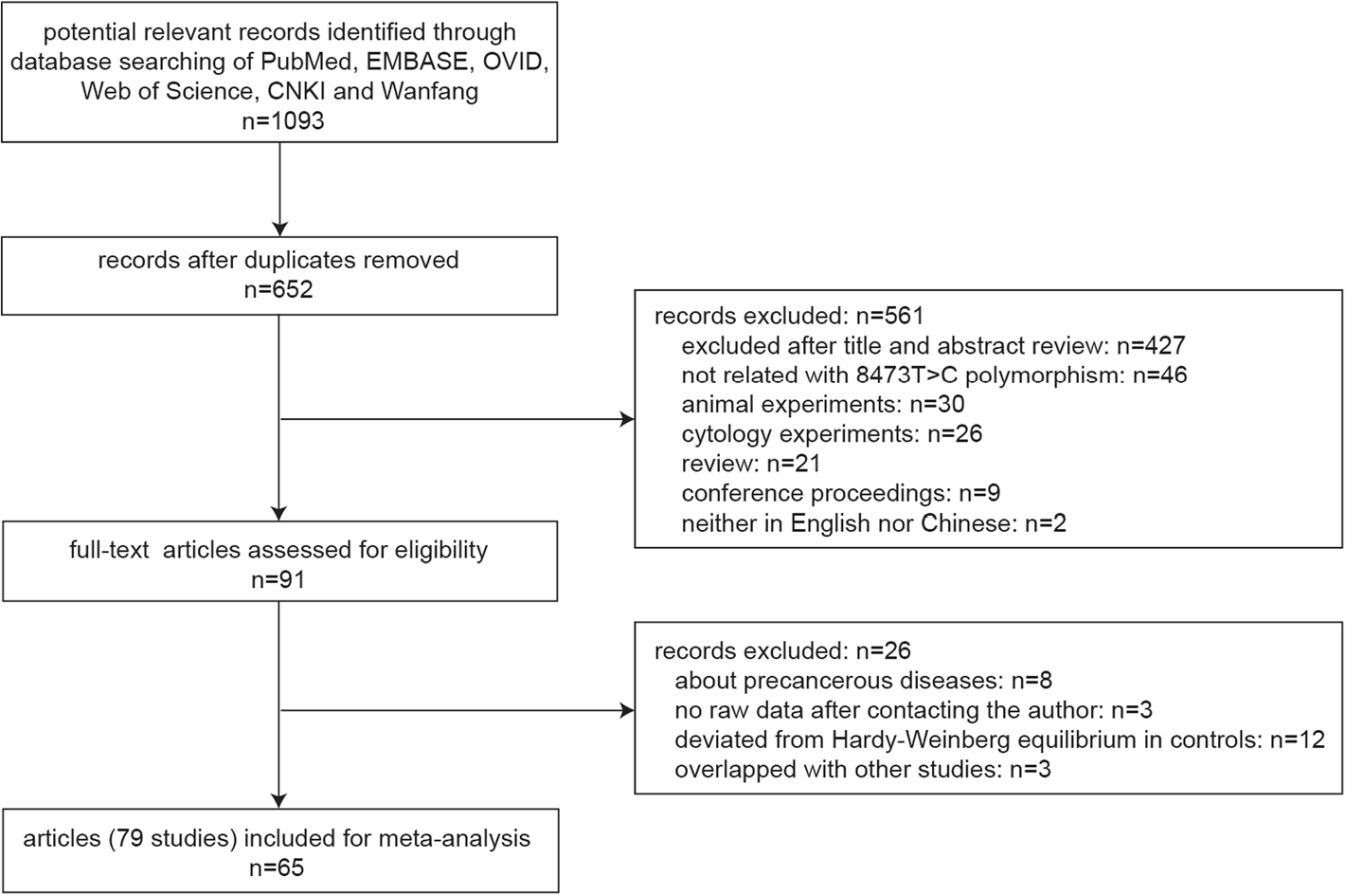
Flow chart of literature search and study selection

A detailed flow chart of included studies is shown in Fig. 1. A systematic search through five electronic databases yielded 652 citations after duplicate removal. After reviewing the titles, abstracts and full texts, articles that were not related with this analysis, meeting, animal or cytology experiments and reviews were removed, leading to the exclusion of 561 publications. The remaining 91 articles were further evaluated for eligibility. Finally, 65 full-text articles (79 studies) that met the inclusion criteria were included in the present meta-analysis.

The primary characteristics of the 79 included studies in this meta-analysis are summarized in Table 1. In our included studies, 38,634 cases and 55,206 controls surveyed the association between *COX-2* 8473 T > C polymorphism and cancer risk. Among these publications, there were 12 colorectal cancer [22–31], 1 ampulla of vater (AV) cancer [32], 4 bladder cancer [33–36], 13 breast cancer [37–46], 2 cervical cancer [47, 48], 1 endometrial cancer [49], 4 esophageal cancer [50–53], 1 extrahepatic bile duct (EHBD) cancer [32], 2 gallbladder cancer [32, 54], 4 gastric cancer [55–58], 1 glioma [59], 2 hepatocellular cancer (HCC) [60, 61], 1 head and neck (HN) cancer [62], 2 laryngeal cancer [50, 63], 11 lung cancer [64–74], 3 nasopharyngeal cancer [50, 75, 76], 3 oral cancer [50, 63, 77], 2 ovarian cancer [78], 1 pancreatic cancer [79], 6 prostate cancer [80–83] and 3 skin cancer [84–86]. Ethnic subgroups were divided into Asian, Caucasian, Australian and African. If it was difficult to distinguish the ethnicity of participants according to content included in the study, ethnicity of the study was termed “Mixed”. Study designs were categorized as PB and HB. The *COX2* 8473 T > C polymorphism was primarily detected by genotyping methods including TaqMan, PCR-RFLP and PCR-PIRA, in addition to the methods of SNPlex, SNP-IT, PCR-KASP, Invader, Illumina GoldenGate, Pyrosequencing and MassARRAY. We used subgroup analysis to search the effects of ethnicity, study design, genotype method and cancer type for the relationship of *COX2* 8473 T > C polymorphism with cancer risk.

**Table 1:** Characteristics of studies included in the meta-analysis

First author	Year	Ethnicity	Country	Control source	Cancer type	Genotype method	cases			controls			HWE	MAF
							TT	TC	CC	TT	TC	CC		
Cox, D.G.	2004	Caucasian	Spain	HB	colorectal	Invader	140	121	29	126	120	25	0.639	0.314
Campa, D.	2004	Caucasian	France	PB	lung	TaqMan	31	107	112	65	99	50	0.304	0.465
Hu, Z.	2005	Asian	China	HB	lung	PCR-PIRA	234	83	5	209	107	7	0.113	0.187
Sorensen, M.	2005	Caucasian	Denmark	PB	lung	TaqMan	127	111	18	115	126	27	0.377	0.336
Campa, D.	2005	Caucasian	France	PB	lung	TaqMan	855	886	224	805	904	228	0.285	0.351
Sakoda, L.C.	2006	Asian	China	PB	AV	TaqMan	30	11	4	541	216	21	0.920	0.166
Gallicchio, L.	2006	Mixed	USA	PB	breast	TaqMan	9	5	0	158	164	34	0.360	0.326
Gallicchio, L.	2006	Mixed	USA	PB	breast	TaqMan	29	26	11	396	416	95	0.353	0.334
Siezen, C.L.	2006	Caucasian	Netherlands	PB	colorectal	Pyrosequencing	97	83	20	190	163	35	0.996	0.300
Siezen, C.L.	2006	Caucasian	Netherlands	PB	colorectal	Pyrosequencing	216	171	55	339	281	73	0.198	0.308
Sakoda, L.C.	2006	Asian	China	PB	EHBD	TaqMan	70	51	5	541	216	21	0.920	0.166
Sakoda, L.C.	2006	Asian	China	PB	gallbladder	TaqMan	165	61	10	541	216	21	0.920	0.166
Park, J.M.	2006	Asian	Korea	HB	lung	PCR-PIRA	352	205	25	330	220	32	0.552	0.244
Shahedi, K.	2006	Caucasian	Sweden	PB	prostate	MassARRAY	571	618	158	306	363	88	0.208	0.356
Cox, D.G.	2007	Mixed	USA	PB	breast	TaqMan	541	567	141	699	808	213	0.383	0.359
Cox, D.G.	2007	Mixed	USA	PB	breast	TaqMan	140	131	30	270	259	81	0.134	0.345
Cox, D.G.	2007	Mixed	USA	PB	breast	TaqMan	281	296	67	278	294	79	0.925	0.347
Gao, J.	2007	Asian	China	HB	breast	PCR-RFLP	404	179	18	429	194	20	0.733	0.182
Vogel, U.	2007	Caucasian	Denmark	PB	breast	PCR-RFLP	167	150	44	155	165	41	0.770	0.342
Lee, T.S.	2007	Asian	Korea	HB	cervical	SNP-IT	115	52	8	101	50	2	0.124	0.176
Campa, D.	2007	Caucasian	France	PB	esophageal	TaqMan	64	84	11	389	377	87	0.756	0.323
Jiang, G.J.	2007	Asian	China	HB	gastric	PCR-PIRA	159	86	9	199	96	9	0.525	0.188
Hou, L.F.	2007	Caucasian	Poland	PB	gastric	TaqMan	137	132	35	165	202	49	0.279	0.361
Campa, D.	2007	Caucasian	France	PB	laryngeal	TaqMan	139	120	22	313	321	77	0.694	0.334
Campa, D.	2007	Caucasian	France	PB	nasopharyngeal	TaqMan	41	47	11	313	321	77	0.694	0.334
Campa, D.	2007	Caucasian	France	PB	oral	TaqMan	72	70	11	313	321	77	0.694	0.334
Cheng, I.	2007	African	USA	HB	prostate	TaqMan	12	39	38	11	49	29	0.162	0.601
Cheng, I.	2007	Caucasian	USA	HB	prostate	TaqMan	183	199	34	196	177	44	0.668	0.318
Lira, M.G.	2007	Caucasian	Italy	HB	skin	PCR-RFLP	44	47	12	64	51	15	0.330	0.312
Vogel, U.	2007	Caucasian	Denmark	PB	skin	TaqMan	123	140	41	145	148	22	0.054	0.305
Yang, H	2008	Mixed	USA	HB	bladder	SNPlex	279	268	76	236	312	85	0.255	0.381
Song, D.K.	2008	Asian	China	HB	bladder	PCR-PIRA	132	39	4	113	61	5	0.337	0.198
Ferguson, H.R.	2008	Caucasian	UK	HB	esophageal	TaqMan	73	106	30	111	113	24	0.537	0.325
Vogel, U.	2008	Caucasian	Denmark	PB	lung	PCR-RFLP	182	183	38	310	341	93	0.959	0.354
Danforth, K.N.	2008	Caucasian	USA	PB	prostate	TaqMan	488	515	143	641	605	137	0.741	0.318
Danforth, K.N.	2008	Caucasian	USA	PB	prostate	TaqMan	517	507	113	501	517	117	0.332	0.331
Abraham, J.E.	2009	Caucasian	UK	PB	breast	TaqMan	927	985	260	996	1010	259	0.903	0.337
Andersen, V	2009	Caucasian	Denmark	PB	colorectal	TaqMan	147	178	34	315	355	95	0.745	0.356
Gong, Z.H	2009	Mixed	USA	PB	colorectal	PCR-RFLP	64	70	28	69	109	33	0.351	0.415
Thompson, C.L.	2009	Caucasian	USA	PB	colorectal	TaqMan	176	189	56	216	199	65	0.081	0.343
Upadhyay, R.	2009	Asian	India	HB	esophageal	PCR-RFLP	63	89	22	81	102	33	0.924	0.389
Srivastava, K.	2009	Asian	India	HB	gallbladder	PCR-RFLP	51	91	25	67	88	29	0.991	0.397
Piranda, D.N.	2010	Mixed	Brazil	PB	breast	TaqMan	125	149	20	120	99	25	0.496	0.305
Dossus, L.	2010	Mixed	Germany	PB	breast	IGG	2697	2664	772	3512	3501	933	0.180	0.338
Pandey, S.	2010	Asian	India	HB	cervical	PCR-RFLP	104	90	6	102	82	16	0.932	0.285
Pereira, C.	2010	Caucasian	Portugal	HB	colorectal	TaqMan	54	51	10	118	114	24	0.638	0.316
Lurie, G.	2010	Mixed	USA	PB	ovarian	TaqMan	169	120	13	338	207	47	0.058	0.254
Lurie, G.	2010	Caucasian	USA	PB	ovarian	TaqMan	333	304	86	490	469	136	0.151	0.338
Gangwar, R.	2011	Asian	India	PB	bladder	PCR-RFLP	82	106	24	97	119	34	0.794	0.374
Brasky, T.M.	2011	Caucasian	USA	PB	breast	TaqMan	432	447	108	732	782	226	0.450	0.355
Akkiz, H.	2011	Caucasian	Turkey	HB	HCC	PCR-RFLP	65	56	8	58	62	9	0.161	0.310
Lim, W.Y.	2011	Asian	Singapore	HB	lung	TaqMan	182	100	15	462	228	28	0.984	0.198
Ozhan, G.	2011	Caucasian	Turkey	HB	pancreatic	PCR-RFLP	74	60	19	71	59	20	0.176	0.330
Mandal, R.K.	2011	Asian	India	HB	prostate	PCR-RFLP	71	86	38	105	113	32	0.853	0.354
Gomez, L.M.	2011	Caucasian	Italy	PB	skin	PCR-RFLP	56	65	17	56	50	18	0.221	0.347
Li, H.Z.	2012	Asian	China	PB	gastric	TaqMan	1048	534	67	1276	568	56	0.450	0.179
Guo, S.J	2012	Asian	China	HB	lung	PCR-RFLP	486	185	15	389	181	32	0.075	0.203
Fawzy, M.S.	2013	Caucasian	Egypt	HB	breast	PCR-RFLP	53	71	36	69	67	14	0.694	0.317
Andersen, V	2013	Caucasian	Denmark	PB	colorectal	PCR-KASP	430	404	97	720	815	203	0.228	0.351
Makar, K.W.	2013	Mixed	USA	PB	colorectal	IGG	851	920	232	1067	1149	333	0.392	0.356
Makar, K.W.	2013	Mixed	USA	PB	colorectal	IGG	552	582	157	887	940	258	0.713	0.349
Ruan, Y.F.	2013	Asian	China	HB	colorectal	PCR-PIRA	98	27	5	80	37	3	0.597	0.179
Song, H.L.	2013	Asian	China	HB	endometrial	PCR-RFLP	68	27	5	69	26	5	0.233	0.180
Lu, Y.J.	2013	Asian	China	HB	esophageal	PCR-RFLP	76	36	7	179	54	5	0.698	0.134
Chang, J.S.	2013	Asian	China	HB	HN	TaqMan	209	89	15	199	86	10	0.850	0.180
Qian, Q.	2014	Asian	China	HB	bladder	TaqMan	4	26	24	1	32	64	0.164	0.825
Gao, J.	2014	Asian	China	HB	breast	TaqMan	299	132	34	515	244	40	0.117	0.203
Vogel, L.K.	2014	Caucasian	Denmark	PB	colorectal	TaqMan	69	87	33	169	191	39	0.156	0.337
Shao, S.S.	2014	Asian	China	HB	HCC	PCR-RFLP	160	92	18	357	164	19	0.975	0.187
Niu, Y.	2014	Asian	China	PB	laryngeal	TaqMan	59	27	4	691	316	25	0.112	0.177
Bhat, I.A.	2014	Asian	India	HB	lung	PCR-RFLP	133	53	4	128	66	6	0.470	0.195
Lan, X.H.	2014	Asian	China	HB	oral	PCR-RFLP	35	14	2	65	32	10	0.053	0.243
Niu, Y.	2014	Asian	China	PB	oral	TaqMan	118	45	5	691	316	25	0.112	0.177
Gao, F.	2015	Asian	China	HB	gastric	TaqMan	171	100	13	193	77	4	0.232	0.155
Lin, R.P.	2015	Asian	China	HB	glioma	TaqMan	129	66	5	109	77	14	0.936	0.263
Cao, Q.	2015	Asian	China	HB	lung	PCR-RFLP	16	19	7	22	25	3	0.233	0.310
Mamoghli, T.	2015	Caucasian	Tunisia	HB	nasopharyngeal	PCR-RFLP	100	80	9	110	99	28	0.433	0.327
Wang, J.L.	2015	Asian	China	HB	nasopharyngeal	PCR-RFLP	139	129	28	110	149	41	0.398	0.385
Moraes, J.L.	2017	Mixed	Brazil	HB	lung	TaqMan	44	43	17	69	106	25	0.107	0.390

*Abbreviations*: *HWE* Hardy-Weinberg equilibrium, *MAF* minor allele frequecy, *HB* hospital based, *PB* population based, *AV* ampulla of vater, *EHBD* extrahepatic bile duct, *HCC* hepatocellular carcinoma, *HN* head and neck, *PCR-RFLP* polymorphism chain reaction restriction fragment length polymorphism, *PCR-PIRA* polymorphism chain reaction based primer-introduced restriction analysis, *PCR-KASP* polymorphism chain reaction based kompetitive allele specific, *IGG* Illumina GoldenGate

### Meta-analysis

#### Overall analysis

The main results of our meta-analysis are listed in Table 2. The association between *COX2* 8473 T > C polymorphism and cancer risk was evaluated in five comparison models: homozygote comparison, heterozygote comparison, dominant model, recessive model and allele analysis. When the homozygote and heterozygote comparisons were carried out, no significant association was found (CC vs.TT: OR = 1.01, 95% CI = 0.93–1.11, *p* = 0.799; TC vs. TT: OR = 0.99, 95% CI = 0.95–1.03, *p* = 0.462). Furthermore, neither dominant nor recessive model discovered significant associations of 8473 T > C polymorphism with cancer risk ((CC + TC) vs. TT: OR = 0.99, 95% CI = 0.95–1.04, *p* = 0.644; CC vs. (TC + TT): OR = 1.01, 95%CI = 0.94–1.09, *p* = 0.779). The allele analysis also didn’t find significant association (C allele vs. T allele: OR = 1.00, 95% CI = 0.96–1.04, *p* = 0.921). Overall, the results of this meta-analysis showed no significant association between *COX-2* 8473 T > C polymorphism and cancer risk.

**Table 2:** Results of overall and stratifed meta-analysis

Genetic model	Group/subgroup	Studies	Heterogeneity test		Statistical model	Test for overall effect	
			I2 (%)	Phet		OR (95% CI)	*P*
CC vs. TT	Overall	79	57.4	0	R	1.01(0.93–1.11)	0.799
	PB	42	58.6	0	R	1.01(0.92–1.11)	0.870
	HB	37	57.3	0	R	1.01(0.83–1.23)	0.915
	Asian	32	55.8	0	R	1.10(0.88–1.37)	0.403
	Caucasian	33	65.9	0	R	1.03(0.90–1.18)	0.652
	Taqman	41	63.9	0	R	1.08(0.94–1.23)	0.272
	PCR-RFLP	23	60.4	0	R	0.94(0.74–1.20)	0.615
	PCR-PIRA	5	0	0.802	F	0.83(0.56–1.23)	0.345
	bladder cancer	4	13.1	0.327	F	***0.74(0.55–0.99)***	***0.040***
	breast cancer	13	53.5	0.012	R	1.01(0.87–1.17)	0.939
	cervical cancer	2	82.6	0.016	R	1.04(0.11–9.53)	0.971
	colorectal cancer	12	17.7	0.270	F	0.95(0.86–1.06)	0.340
	esophageal cancer	4	61.1	0.052	R	1.30(0.72–2.33)	0.390
	gallbladder cancer	2	0	0.532	F	1.28(0.78–2.12)	0.326
	gastric cancer	4	52.4	0.098	R	1.34(0.85–2.13)	0.210
	HCC	2	59.9	0.114	F	1.54(0.88–2.70)	0.128
	laryngeal cancer	2	67.3	0.080	R	0.98(0.35–2.75)	0.973
	lung cancer	11	80.5	0	R	0.97(0.65–1.45)	0.883
	nasopharyngeal cancer	3	56.1	0.103	F	***0.59(0.40–0.86)***	***0.007***
	oral cancer	3	0	0.404	F	0.68(0.40–1.16)	0.158
	ovarian cancer	2	51.6	0.151	F	0.84(0.64–1.10)	0.205
	prostate cancer	6	42.8	0.120	F	1.10(0.95–1.28)	0.192
	skin cancer	3	42.6	0.175	F	***1.51(1.02–2.25)***	***0.041***
TC vs. TT	Overall	79	33.1	0.003	R	0.99(0.95–1.03)	0.462
	PB	42	28.4	0.047	R	1.00(0.96–1.04)	0.908
	HB	37	37.7	0.012	R	0.96(0.88–1.04)	0.303
	Asian	32	43.4	0.005	R	0.98(0.90–1.07)	0.675
	Caucasian	33	23.1	0.119	F	0.99(0.95–1.04)	0.679
	Taqman	41	36.2	0.012	R	1.03(0.97–1.09)	0.313
	PCR-RFLP	23	11.6	0.303	F	0.97(0.90–1.05)	0.494
	PCR-PIRA	5	50.4	0.089	R	***0.78(0.61–0.99)***	***0.037***
	bladder cancer	4	49.4	0.115	F	***0.75(0.62–0.90)***	***0.002***
	breast cancer	13	0	0.540	F	0.99(0.94–1.04)	0.676
	cervical cancer	2	0	0.604	F	1.00(0.74–1.37)	0.980
	colorectal cancer	12	3.8	0.408	F	0.97(0.90–1.03)	0.305
	esophageal cancer	4	0	0.772	F	***1.35(1.10–1.66)***	***0.004***
	gallbladder cancer	2	41.6	0.191	F	1.05(0.80–1.38)	0.706
	gastric cancer	4	57.2	0.071	R	1.10(0.89–1.36)	0.389
	HCC	2	52.2	0.148	F	1.11(0.85–1.44)	0.467
	laryngeal cancer	2	0	0.542	F	0.88(0.69–1.13)	0.322
	lung cancer	11	51.3	0.025	R	0.90(0.79–1.03)	0.140
	nasopharyngeal cancer	3	33.3	0.223	F	0.84(0.67–1.06)	0.135
	oral cancer	3	0	0.867	F	0.88(0.69–1.12)	0.307
	ovarian cancer	2	14.7	0.279	F	1.02(0.86–1.20)	0.855
	prostate cancer	6	3.1	0.397	F	1.02(0.93–1.12)	0.662
	skin cancer	3	0	0.806	F	1.20(0.93–1.54)	0.154
(CC + TC) vs. TT	Overall	79	50.0	0	R	0.99(0.95–1.04)	0.644
	PB	42	46.4	0.001	R	1.00(0.95–1.05)	0.992
	HB	37	53.9	0	R	0.97(0.88–1.06)	0.490
	Asian	32	57.0	0	R	0.99(0.90–1.10)	0.892
	Caucasian	33	51.5	0	R	1.01(0.95–1.08)	0.775
	Taqman	41	53.3	0	R	1.04(0.97–1.11)	0.249
	PCR-RFLP	23	43.4	0.015	R	0.98(0.88–1.10)	0.758
	PCR-PIRA	5	48.8	0.099	R	***0.79(0.63–0.98)***	***0.035***
	bladder cancer	4	52.9	0.095	R	0.73(0.53–1.00)	0.052
	breast cancer	13	19.0	0.251	F	1.00(0.95–1.04)	0.877
	cervical cancer	2	0	0.862	F	0.98(0.73–1.32)	0.909
	colorectal cancer	12	4.3	0.403	F	0.96(0.90–1.03)	0.237
	esophageal cancer	4	0	0.414	F	***1.33(1.10–1.63)***	***0.004***
	gallbladder cancer	2	2.2	0.312	F	1.08(0.84–1.40)	0.557
	gastric cancer	4	65.6	0.033	R	1.13(0.90–1.42)	0.300
	HCC	2	67.1	0.081	R	1.08(0.66–1.77)	0.764
	laryngeal cancer	2	7.3	0.299	F	0.87(0.68–1.10)	0.238
	lung cancer	11	72.7	0	R	0.92(0.78–1.10)	0.363
	nasopharyngeal cancer	3	47.0	0.152	F	***0.79(0.64–0.98)***	***0.030***
	oral cancer	3	0	0.856	F	0.85(0.67–1.08)	0.180
	ovarian cancer	2	0	0.565	F	0.98(0.84–1.14)	0.784
	prostate cancer	6	21.0	0.275	F	1.04(0.95–1.13)	0.408
	skin cancer	3	0	0.979	F	1.25(0.99–1.59)	0.063
CC vs. (TC + TT)	Overall	79	52.6	0	R	1.01(0.94–1.09)	0.779
	PB	42	53.2	0	R	1.01(0.93–1.09)	0.831
	HB	37	53.3	0	R	1.01(0.85–1.21)	0.876
	Asian	32	52.9	0	R	1.07(0.86–1.32)	0.500
	Caucasian	33	58.5	0	R	1.02(0.91–1.14)	0.715
	Taqman	41	60.9	0	R	1.05(0.94–1.18)	0.400
	PCR-RFLP	23	55.3	0.001	R	0.94(0.76–1.17)	0.572
	PCR-PIRA	5	0	0.845	F	0.88(0.59–1.30)	0.510
	bladder cancer	4	25.9	0.256	F	0.78(0.61–1.01)	0.061
	breast cancer	13	53.4	0.012	R	1.01(0.88–1.16)	0.884
	cervical cancer	2	83.8	0.013	R	1.04(0.11–10.14)	0.972
	colorectal cancer	12	19.1	0.256	F	0.97(0.88–1.06)	0.471
	esophageal cancer	4	60.8	0.054	R	1.12(0.64–1.95)	0.695
	gallbladder cancer	2	13.5	0.282	F	1.13(0.71–1.80)	0.615
	gastric cancer	4	27.8	0.245	F	1.30(1.00–1.68)	0.052
	HCC	2	42.5	0.187	F	1.51(0.87–2.61)	0.141
	laryngeal cancer	2	62.6	0.102	F	0.80(0.51–1.26)	0.338
	lung cancer	11	75.4	0	R	0.99(0.70–1.38)	0.932
	nasopharyngeal cancer	3	46.9	0.152	F	***0.65(0.46–0.94)***	***0.020***
	oral cancer	3	0	0.388	F	0.71(0.42–1.18)	0.182
	ovarian cancer	2	65.5	0.088	R	0.75(0.42–1.34)	0.336
	prostate cancer	6	44.6	0.108	F	1.11(0.97–1.27)	0.137
	skin cancer	3	57.6	0.095	R	1.01(0.94–1.09)	0.454
C allele vs. T allele	Overall	79	62.0	0	R	1.00(0.96–1.04)	0.921
	PB	42	59.9	0	R	1.01(0.96–1.05)	0.810
	HB	37	64.8	0	R	0.98(0.90–1.07)	0.656
	Asian	32	66.4	0	R	1.00(0.91–1.09)	0.956
	Caucasian	33	66.9	0	R	1.02(0.96–1.08)	0.573
	Taqman	41	66.5	0	R	1.04(0.98–1.10)	0.239
	PCR-RFLP	23	61.4	0	R	0.99(0.89–1.09)	0.794
	PCR-PIRA	5	39.9	0.155	F	***0.84(0.74–0.96)***	***0.010***
	bladder cancer	4	57.4	0.070	R	***0.76(0.60–0.96)***	***0.020***
	breast cancer	13	47.8	0.028	R	1.00(0.94–1.06)	0.938
	cervical cancer	2	9.5	0.293	F	0.95(0.75–1.22)	0.699
	colorectal cancer	12	12.8	0.319	F	0.97(0.93–1.02)	0.222
	esophageal cancer	4	56.6	0.075	R	1.21(0.96–1.52)	0.100
	gallbladder cancer	2	0	0.759	F	1.07(0.88–1.31)	0.496
	gastric cancer	4	67.7	0.026	R	1.14(0.94–1.38)	0.195
	HCC	2	73.4	0.052	R	1.10(0.71–1.71)	0.658
	laryngeal cancer	2	47.3	0.168	F	0.88(0.73–1.06)	0.183
	lung cancer	11	83.0	0	R	0.96(0.82–1.14)	0.661
	nasopharyngeal cancer	3	54.1	0.113	F	***0.80(0.68–0.94)***	***0.007***
	oral cancer	3	0	0.669	F	0.85(0.70–1.03)	0.106
	ovarian cancer	2	0	0.850	F	0.95(0.85–1.07)	0.428
	prostate cancer	6	44.2	0.111	F	1.05(0.98–1.12)	0.188
	skin cancer	3	0	0.589	F	***1.21(1.02–1.45)***	***0.031***

*Abbreviations*: *OR* odds ratios, *CI* confidence intervals, *R* random effects model, *F* fixed effects model, *HB* hospital based, *PB* population based, *PCR-RFLP* polymorphism chain reaction restriction fragment length polymorphism, *PCR-PIRA* polymorphism chain reaction based primer-introduced restriction analysis, *HCC* hepatocellular carcinoma

The results are in bold italic if *P <0.05*

#### Subgroup analysis

In order to estimate the effects of specific study characteristics on the relationship between *COX-2* 8473 T > C polymorphism and cancer risk, we carried out subgroup analysis in control source, ethnicity, genotyping method and type of cancer under a variety of genetic models. For control source subgroup, whether the source of controls was population-based (PB) or hospital-based (HB), no association between 8473 T > C polymorphism and cancer risk was found. When stratified according to ethnicity, we observed no significant associations in Asians or Caucasians. Stratified by genotyping method, no relationship was detected in TaqMan and PCR-RFLP. However, by comparison, we discovered statistically significant decreased cancer risk in PCR-PIRA (TC vs. TT: OR = 0.78, 95% CI: 0.61–0.99, *p* = 0.037; (CC + TC) vs. TT: OR = 0.79, 95% CI: 0.63–0.78, *P* = 0.035; C allele vs. T allele: OR = 0.84, 95% CI: 0.74–0.96, *P* = 0.010). According to cancer type, 8473 T > C polymorphism was associated with a statistically significant decreased risk for nasopharyngeal cancer except for heterozygote comparison (CC vs. TT: OR = 0.59, 95% CI: 0.40–0.86, *P* = 0.007; (CC + TC) vs. TT: OR = 0.79, 95% CI: 0.64–0.98, *P* = 0.030; CC vs. (TC + TT): OR = 0.65, 95%CI: 0.46–0.94, *P* = 0.020; C allele vs. T allele: OR = 0.80, 95% CI: 0.68–0.94, *P* = 0.007). In the group with bladder cancer, we also found a decreased risk in the homozygote comparison, heterozygote comparison and allele analysis (CC vs. TT: OR = 0.74, 95% CI = 0.55–0.99, *P* = 0.040; TC vs. TT: OR = 0.75, 95% CI = 0.62–0.90, *P* = 0.002; C allele vs. T allele: OR = 0.76, 95% CI = 0.60–0.96, *P* = 0.020), but not in the dominant model and recessive model. However, for the esophageal cancer group, the *COX-2* 8473 T > C polymorphism was significantly associated with an increased risk in the heterozygote comparison and dominant model (TC vs. TT: OR = 1.35, 95% CI = 1.10–1.66, *P* = 0.004; (CC + TC) vs. TT: OR = 1.33, 95% CI = 1.10–1.63, *P* = 0.004), but not in the homozygote comparison, recessive model and allele analysis. For the group of skin cancer, we also observed the association of a significantly increased risk in the homozygote comparison and allele analysis (CC vs. TT: OR = 1.51, 95% CI = 1.02–2.25, *P* = 0.041; C allele vs. T allele: OR = 1.21, 95% CI = 1.02–1.45, *P* = 0.031, respectively), but not in heterozygote comparison, dominant model and recessive model. On the contrary, the result of breast cancer indicated no relationship with this polymorphism. Similarly, we also observed no significant association of 8473 T > C polymorphism with other cancers, including cervical cancer, colorectal cancer, gallbladder cancer, gastric cancer, HCC, lung cancer, oral cancer, ovarian cancer and prostate cancer. The detailed results were shown in Table 2.

#### Test of heterogeneity and sensitivity analysis

Significant heterogeneity was obvious in all the comparisons of *COX-2* 8473 T > C polymorphism (Table 2). Studies were excluded one by one to evaluate their influence on the test of heterogeneity and the credibility of our results. The results revealed that the corresponding pooled ORs and 95% CIs were not changed (Additional file 2: Figure S1, Additional file 3: Figure S2, Additional file 4: Figure S3 and Additional file 5: Figure S4), implying that the results of the present meta-analysis were credible and robust.

#### Publication bias

**Fig. 2:**
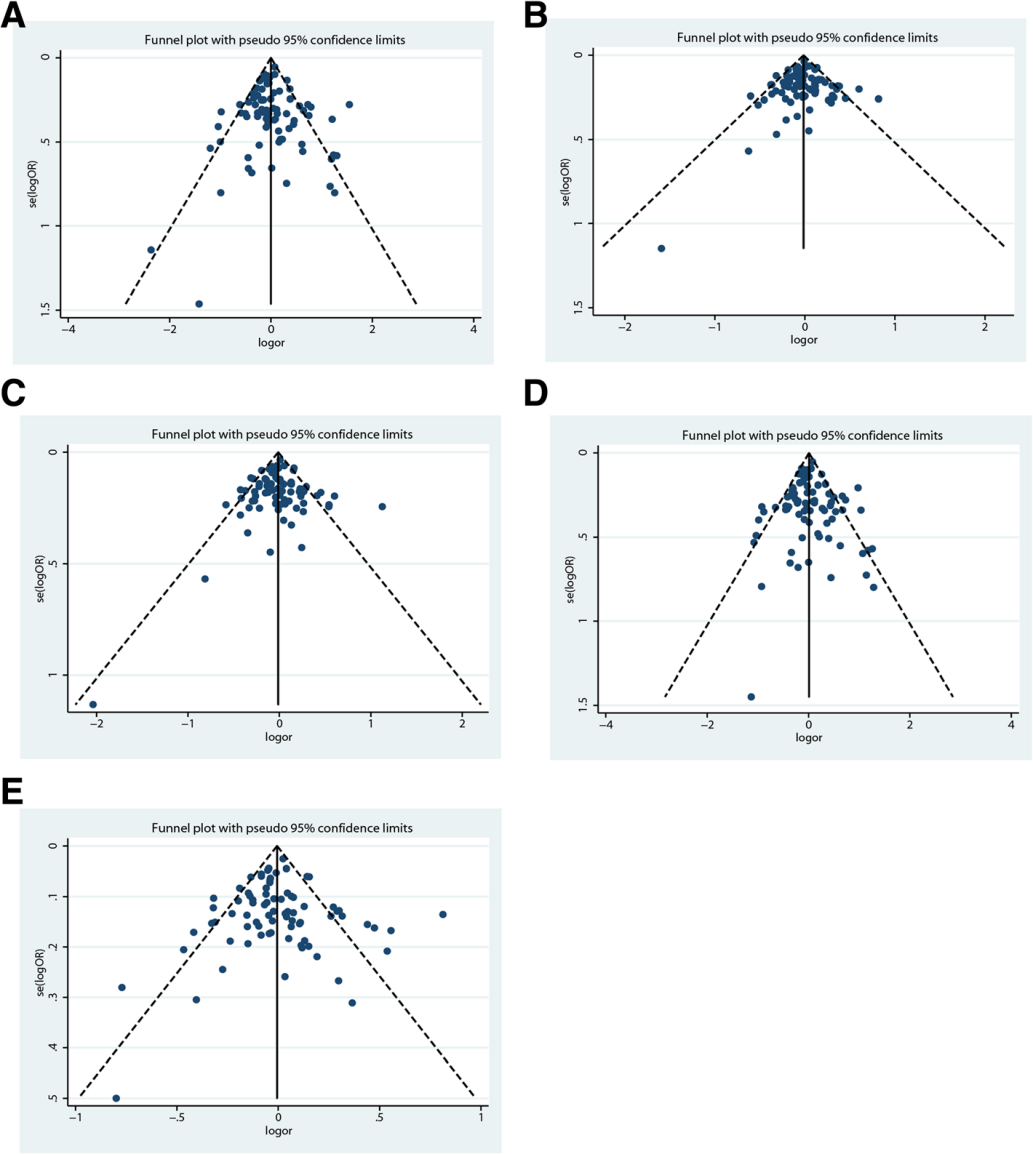
**a**. Funnel plots for the publication bias test in the overall analysis under homozygote comparison. **b**. Funnel plots for the publication bias test in the overall analysis under heterozygote comparison. **c**. Funnel plots for the publication bias test in the overall analysis under dominant model. **d**. Funnel plots for the publication bias test in the overall analysis under recessive model. **e**. Funnel plots for the publication bias test in the overall analysis under allele analysis

The Begg’s and Egger’s tests were performed to quantitatively assess the publication bias of this meta-analysis. *P* < 0.05 observed in the allelic genetic models was considered representive of statistically significant publication bias. The P details for bias were presented in Table 3. There was no significant publication bias in the overall analysis under each model. Moreover, the funnel plots quantitatively evaluating the publication bias did not reveal any evidence of obvious asymmetry in any model (Fig. 2).

**Table 3:** Results of publication bias test

Compared genotype	Begg’s test		Egger’s test	
	z value	*P* value	t value	*P* value
CC vs. TT	1.10	0.273	0.34	0.734
TC vs. TT	−0.16	0.876	−0.14	0.890
(CC + TC) vs. TT	0.64	0.523	0.06	0.951
CC vs. (TC + TT)	0.93	0.354	0.24	0.807
C allele vs. T allele	0.79	0.429	0.14	0.891

*P* value < 0.05 was considered as significant publication bias

#### Trial sequential analysis (TSA) results

**Fig. 3:**
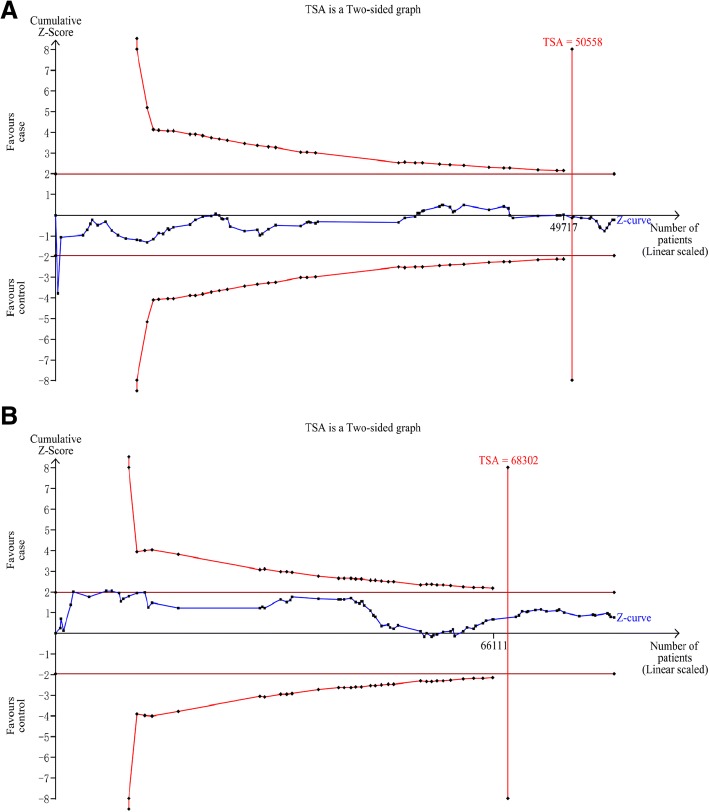
**a**. TSA for overall analysis under homozygote comparison. **b**. TSA for overall analysis under heterozygote comparison. The required information size was calculated based on a two side α = 5%, β = 20% (power 80%), and an anticipated relative risk reduction of 10%

As shown in Fig. 3, in order to prove the conclusions, the sample size required in the overall analysis was 50,558 cases for homozygote comparison, and 68,302 cases for heterozygote comparison. The results showed that the cumulative Z-cure didn’t exceed the TSA boundary, but the total number of cases and controls exceeded the required sample size, indicating that adequate evidence of our conclusions were established and no further relevant trials were needed.

## Discussion

Inflammation has been considered as an acting element for the pathogenesis of cancer. Prostaglandins are important molecules in the inflammatory response, and they are produced from arachidonic aid through the catalytic activity of COX-2. COX-2 cannot be detected under normal conditions, but rapidly induced in response to various inflammatory stimulus [7]. The expression level of *COX-2* gene is regulated by a series of regulatory elements located in *COX-2* promoter region, including nuclear factor-κb(NF- κB)/nuclear factor interleukin-6 (NF-IL6)/CCAAT/enhancer-binding protein (C/EBP) binding sites, cyclic AMP-response element (CRE) and activation protein 1 (AP-1) [87]. Further studies indicated that 3’UTR of *COX-2* gene of murine also contains several regulatory elements affecting the stability of mRNA and the efficiency of translation [12], which played vital roles in stabilization, degradation, and translation of the transcripts [88, 89]. According to the above studies, many researchers hypothesized that polymorphism sites in 3’UTR of *COX-2* gene, with 8473 T > C polymorphism included, might increase the expression of *COX-2* and affect the susceptibility of cancer. Therefore, the correlation between 8473 T > C polymorphism in 3’UTR of *COX-2* gene and cancer susceptibility has been of great interest in polymorphism research. In this meta-analysis, not only did we try to make sure whether 8473 T > C polymorphism has any relationship with the susceptibility of overall cancer, but we also performed TSA to efficiently decrease the risk of type I error and evaluate whether our results were stable.

In the present meta-analysis, we comprehensively researched the association of the 8473 T > C polymorphism in the 3’UTR region of *COX-2* with cancer risk in all population through 79 studies. The results showed that no significant association between 8473 T > C polymorphism we studied and overall cancer risk was detected under all five genetic comparisons. However, we discovered significant heterogeneity among studies, therefore, further sensitivity analyses were conducted. Though the studies were eliminated one by one, heterogeneity remained significant. Moreover, several subgroup analyses, performed according to control source, ethnicity, genotyping method and type of cancer in all compared genetic models, could not explain the source of heterogeneity. In control source subgroup, no statistical significance association was found neither in PB nor HB. For ethnicity subgroup, whether in Asians or Caucasians, the polymorphism had no influence on cancer risk. The results might indicate that different individuals in the studies have the same risk to cancer. Moreover, only in the subgroup of PCR-PIRA, 8473 T > C polymorphism was linked to decrease risk to overall cancer in heterozygote comparison, recessive model and allele analysis, suggesting that different genotype detecting methods used in studies might influence the results. In the stratification analysis by type of cancer, the results indicated that the 8473 T > C polymorphism was associated with a statistically significant decreased risk for nasopharyngeal cancer in other four models except for heterozygote comparison, and bladder cancer in the homozygote comparison, heterozygote comparison and allele analysis. However, we observed an increased risk for esophageal cancer in heterozygote comparison and dominant model, and for skin cancer in homozygote comparison and allele analysis. The factors that contributed to this contradiction might include the following three aspects. Firstly, inconsistent results might be attributed to the different pathogenesis of the cancer. Secondly, 8473 T > C polymorphism might play different roles in different cancers. Most importantly, the influence of *COX-2* gene 8473 T > C polymorphism on cancer risk might be affected by complex interactions between gene and environment. For example, smoking, the most important risk factor of lung cancer, could induce *COX-2* expression [90].

Currently, some meta-analysis have investigated the relationship of 8473 T > C polymorphism with susceptibility to some types of cancer. Interestingly, part of the previous studies found some strong associations inconsistent with the result of our meta-analysis. Such as the report by Liu et al. [91] indicated that *COX-2* gene 8473 T > C polymorphism was a factor for suffering from lung cancer, and Zhu et al. [92] suggested that 8473 T > C polymorphism might cause a decreased risk of lung cancer. Like Pan et al. [93], the current study supports the view that no significant association between 8473 T > C polymorphism and lung cancer risk. The reasons for this result may be as follows, firstly, the quality of original studies directly influences the reliability of the meta-analysis. In our meta-analysis the quality assessment of all the studies related with cancer was performed by using NOS, and low-quality studies were excluded. Secondly, the studies with the most recent or larger sample size were included, we therefore carried out a more systematic review of all eligible studies on the COX-2 8473 T > C polymorphisms and risk of lung cancer. Thirdly, the result of this polymorphism on cancer susceptibility might be influenced by some environmental factors or other polymorphisms, such as smoking. Meanwhile, some significant correlations we found were not shown in previous meta-analysis. For example, 8473 T > C polymorphism was associated with a decreased risk in nasopharyngeal cancer. When later studies were included in the meta-analysis, the contradiction didn’t appear, suggesting that the conclusions of previous meta-analysis with less number of studies might be reliable. More studies are required to achieve a more reliable result.

Obviously, we clarified the association in this meta-analysis, including more studies with the larger information size. Besides, it is the first TSA that comprehensively elaborated the influence of *COX-2* 8473 T > C polymorphism in response to cancer risk. However, several limitations should be taken into consideration in this meta-analysis. To begin with, only publications written in English or Chinese were included in our analysis. Therefore, selection bias might be inevitable. Secondly, there was significant heterogeneity in this meta-analysis between the polymorphism and cancer under all five genetic models. Moreover, the source of heterogeneity could not be explained by using subgroup and sensitivity analysis. Finally, as a complicated disease, the pathogenesis of cancer is strongly associated with environmental factors and the interactions with multifarious genetic factors rather than the effect of any single gene. Therefore, gene-to-environment interactions play a vital role in evaluating genetic polymorphisms. More original studies are required to estimate potential interactions between gene and gene, as well as gene and environment.

## Conclusions

The results of this meta-analysis manifested that the association between *COX-2* 8473 T > C polymorphism and overall cancer was not detected under all five genetic comparisons. In the stratification analysis of cancer type, 8473 T > C polymorphism might be associated with a statistically significant decreased risk for nasopharyngeal cancer and bladder cancer, but an increased risk for esophageal cancer and skin cancer. And most importantly, in order to verify the conclusions of this analysis, further studies are needed to assess the potential gene-gene and gene-environment interactions.

## Additional files

Additional file 1: **Table S1.** Results of Newcastle–Ottawa scale (NOS) assessment for the included studies. (DOCX 23 kb)

Additional file 2: **Figure S1.** A. Sensitivity analysis of 8473 T > C polymorphism and cancer risk in HB subgroup under homozygote comparison. B. Sensitivity analysis of 8473 T > C polymorphism and cancer risk in PB subgroup under homozygote comparison. (TIF 4832 kb)

Additional file 3: **Figure S2.** A. Sensitivity analysis of 8473 T > C polymorphism and cancer risk in Asians under homozygote comparison. B. Sensitivity analysis of 8473 T > C polymorphism and cancer risk in Caucasians under homozygote comparison. (TIF 4809 kb)

Additional file 4: **Figure S3.** A. Sensitivity analysis of 8473 T > C polymorphism and cancer risk in TaqMan under homozygote comparison. B. Sensitivity analysis of 8473 T > C polymorphism and cancer risk in PCR-RFLP under homozygote comparison. (TIF 4661 kb)

Additional file 5: **Figure S4.** A. Sensitivity analysis of 8473 T > C polymorphism and cancer risk in breast cancer under homozygote comparison. B. Sensitivity analysis of 8473 T > C polymorphism and cancer risk in lung cancer under homozygote comparison. (TIF 4758 kb)

## Abbreviations

AV: Ampulla of vater
CI: Confidence intervals
EHBD: Extrahepatic bile duct
F: Fixed effects model
HB: Hospital based
HCC: Hepatocellular carcinoma
HN: Head and neck
HWE: Hardy-Weinberg equilibrium
IGG: Illumina GoldenGate
MAF: Minor allele frequecy
OR: Odds ratios
PB: Population based
PCR-KASP: Polymorphism chain reaction based kompetitive allele specific
PCR-PIRA: Polymorphism chain reaction based primer-introduced restriction analysis
PCR-RFLP: Polymorphism chain reaction restriction fragment length polymorphism
R: Random effects model
