# Supplementary material for: Association between cyclooxygenase-2 (COX-2) 8473 T > C polymorphism and cancer risk: a meta-analysis and trial sequential analysis
Source: BMC Cancer. 2018 Aug 24;18:847. doi: 10.1186/s12885-018-4753-3 (PMC6109290; doi:10.1186/s12885-018-4753-3)
Supplement: Supplementary file 1 — Table S1. Results of Newcastle–Ottawa scale (NOS) assessment for the included studies. (DOCX 23 kb) [file 12885_2018_4753_MOESM1_ESM.docx]

| **Table S1. Results of Newcastle–Ottawa scale (NOS) assessment for the included studies.** | | | | | |
| --- | --- | --- | --- | --- | --- |
| **First author** | **Year** | **Selection** | **Comparability** | **Exposure** | **Total NOS star rating** |
| Cox,D.G. | 2004 | 3 | 2 | 2 | 7 |
| Campa,D. | 2004 | 3 | 2 | 3 | 8 |
| Hu,Z. | 2005 | 3 | 2 | 2 | 7 |
| Sorensen,M. | 2005 | 4 | 2 | 2 | 8 |
| Campa,D. | 2005 | 3 | 2 | 2 | 7 |
| Sakoda,L.C. | 2006 | 3 | 2 | 2 | 7 |
| Gallicchio,L. | 2006 | 3 | 2 | 3 | 8 |
| Siezen,C.L. | 2006 | 3 | 2 | 3 | 8 |
| Park,J.M. | 2006 | 3 | 2 | 2 | 7 |
| Shahedi,K. | 2006 | 3 | 2 | 2 | 7 |
| Cox,D.G. | 2007 | 3 | 2 | 2 | 7 |
| Gao,J. | 2007 | 4 | 2 | 2 | 8 |
| Vogel,U. | 2007 | 3 | 2 | 2 | 7 |
| Lee,T.S. | 2007 | 4 | 1 | 2 | 7 |
| Jiang,G.J. | 2007 | 3 | 2 | 2 | 7 |
| Hou,L.F. | 2007 | 2 | 2 | 2 | 6 |
| Cheng,I. | 2007 | 3 | 2 | 1 | 6 |
| Lira,M.G. | 2007 | 3 | 2 | 2 | 7 |
| Vogel,U. | 2007 | 3 | 2 | 2 | 7 |
| Campa,D. | 2007 | 3 | 2 | 2 | 7 |
| Yang,H | 2008 | 3 | 2 | 1 | 6 |
| Ferguson,H.R. | 2008 | 4 | 2 | 3 | 9 |
| Vogel,U. | 2008 | 3 | 2 | 2 | 7 |
| Danforth,K.N. | 2008 | 3 | 1 | 2 | 6 |
| Song,D.K. | 2008 | 4 | 2 | 2 | 8 |
| Andersen,V | 2009 | 3 | 2 | 2 | 7 |
| Gong,Z.H | 2009 | 2 | 2 | 2 | 6 |
| Thompson,C.L. | 2009 | 2 | 2 | 2 | 6 |
| Upadhyay,R. | 2009 | 4 | 2 | 2 | 8 |
| Srivastava,K. | 2009 | 4 | 2 | 1 | 7 |
| Abraham,J.E. | 2009 | 3 | 1 | 2 | 6 |
| **First author** | **Year** | **Selection** | **Comparability** | **Exposure** | **Total NOS star rating** |
| Piranda,D.N. | 2010 | 3 | 2 | 2 | 7 |
| Pandey,S. | 2010 | 4 | 2 | 2 | 8 |
| Pereira,C. | 2010 | 4 | 2 | 2 | 8 |
| Lurie,G. | 2010 | 3 | 2 | 2 | 7 |
| Dossus,L. | 2010 | 3 | 2 | 2 | 7 |
| Gangwar,R. | 2011 | 3 | 2 | 2 | 7 |
| Brasky,T.M. | 2011 | 3 | 2 | 2 | 7 |
| Akkiz, H. | 2011 | 3 | 2 | 2 | 7 |
| Lim,W.Y. | 2011 | 3 | 1 | 2 | 6 |
| Ozhan,G. | 2011 | 3 | 2 | 2 | 7 |
| Mandal,R.K. | 2011 | 3 | 2 | 2 | 7 |
| Gomez,L.M. | 2011 | 3 | 1 | 2 | 6 |
| Li,H.Z. | 2012 | 4 | 2 | 2 | 8 |
| Guo,S.J | 2012 | 3 | 1 | 2 | 6 |
| Fawzy,M.S. | 2013 | 2 | 2 | 2 | 6 |
| Andersen,V | 2013 | 3 | 2 | 2 | 7 |
| Makar,K.W. | 2013 | 3 | 2 | 3 | 8 |
| Song,H.L. | 2013 | 3 | 1 | 2 | 6 |
| Lu,Y.J. | 2013 | 4 | 2 | 2 | 8 |
| Ruan,Y.F. | 2013 | 3 | 2 | 2 | 7 |
| Chang,J.S. | 2013 | 3 | 2 | 2 | 7 |
| Qian,Q. | 2014 | 3 | 2 | 2 | 7 |
| Gao,J. | 2014 | 4 | 1 | 2 | 7 |
| Vogel,L.K. | 2014 | 3 | 1 | 2 | 6 |
| Shao,S.S. | 2014 | 3 | 2 | 2 | 7 |
| Bhat,I.A. | 2014 | 4 | 2 | 2 | 8 |
| Niu,Y. | 2014 | 3 | 2 | 2 | 7 |
| Lan,X.H. | 2014 | 3 | 2 | 2 | 7 |
| Lin,R.P. | 2015 | 3 | 2 | 1 | 6 |
| Mamoghli,T. | 2015 | 4 | 2 | 3 | 9 |
| Wang,J.L. | 2015 | 3 | 2 | 2 | 7 |
| Cao,Q | 2015 | 3 | 2 | 2 | 7 |
| **First author** | **Year** | **Selection** | **Comparability** | **Exposure** | **Total NOS star rating** |
| Gao,F. | 2015 | 3 | 2 | 2 | 7 |
| Moraes,J.L. | 2017 | 3 | 1 | 2 | 6 |
