# Supplementary figures and images for: Association between cyclooxygenase-2 (COX-2) 8473 T > C polymorphism and cancer risk: a meta-analysis and trial sequential analysis
Source: BMC Cancer. 2018 Aug 24;18:847. doi: 10.1186/s12885-018-4753-3 (PMC6109290; doi:10.1186/s12885-018-4753-3)

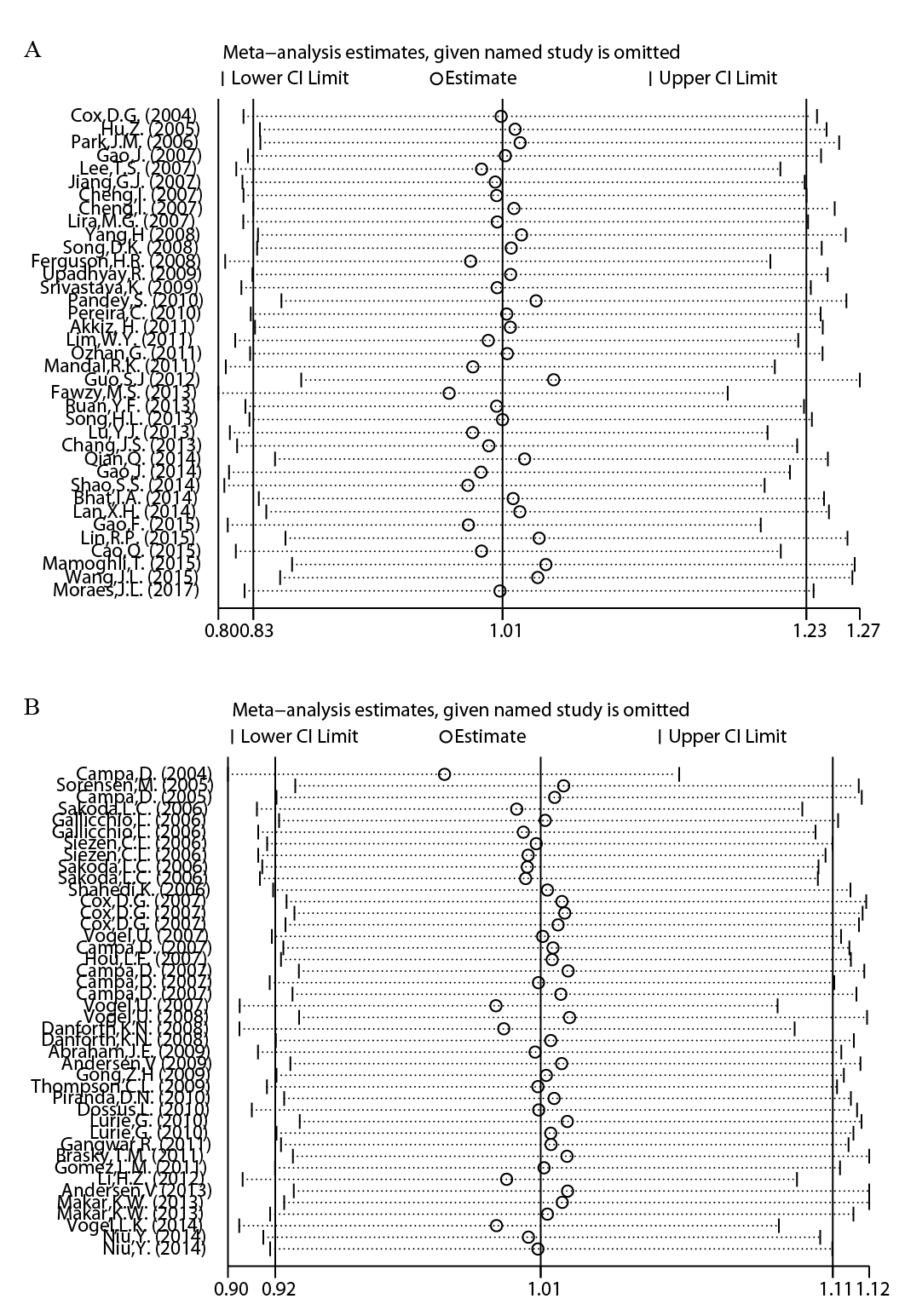

Supplement: Supplementary file 2 — Figure S1. A. Sensitivity analysis of 8473 T > C polymorphism and cancer risk in HB subgroup under homozygote comparison. B. Sensitivity analysis of 8473 T > C polymorphism and cancer risk in PB subgroup under homozygote comparison. (TIF 4832 kb) [file 12885_2018_4753_MOESM2_ESM.tif]

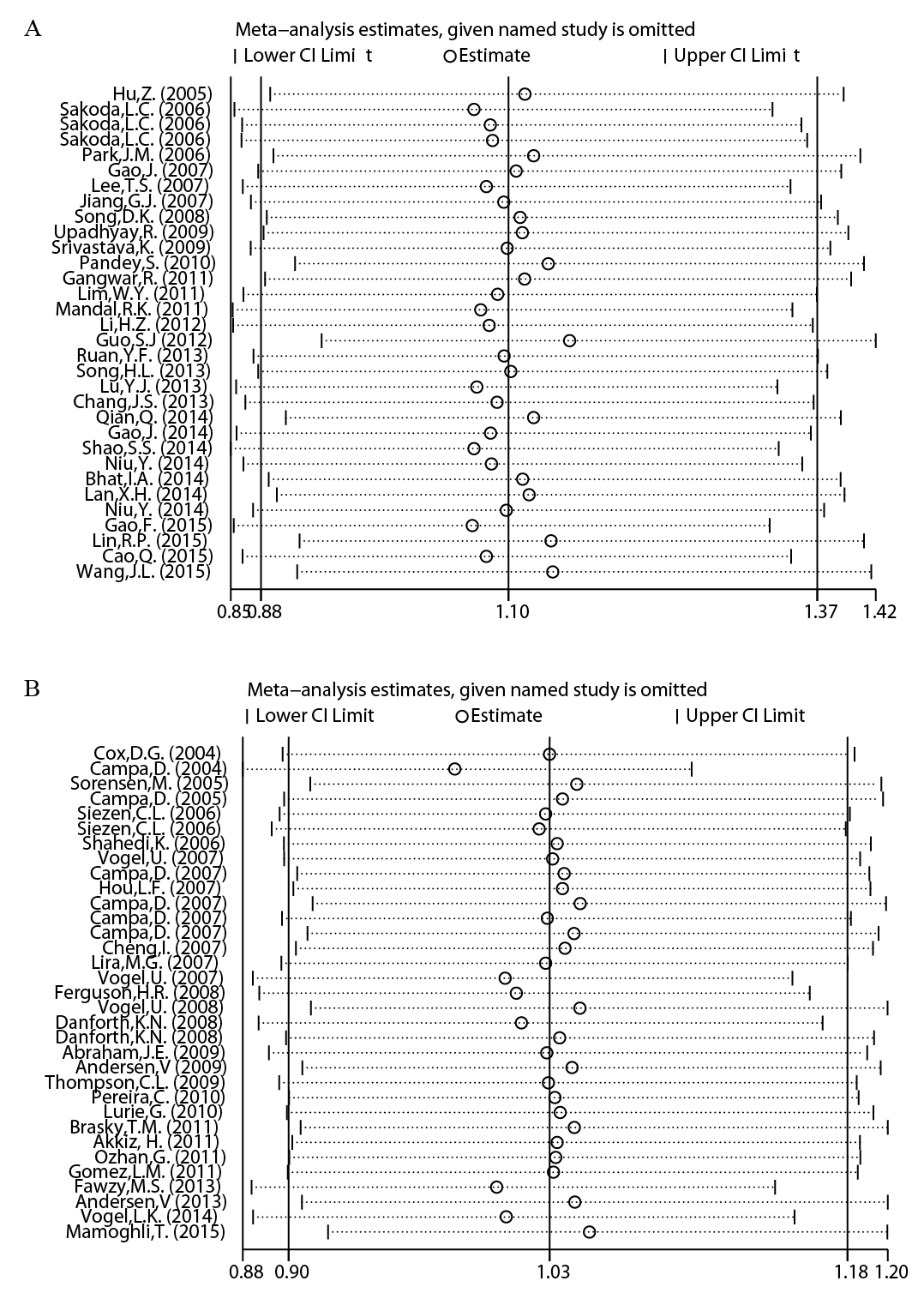

Supplement: Supplementary file 3 — Figure S2. A. Sensitivity analysis of 8473 T > C polymorphism and cancer risk in Asians under homozygote comparison. B. Sensitivity analysis of 8473 T > C polymorphism and cancer risk in Caucasians under homozygote comparison. (TIF 4809 kb) [file 12885_2018_4753_MOESM3_ESM.tif]

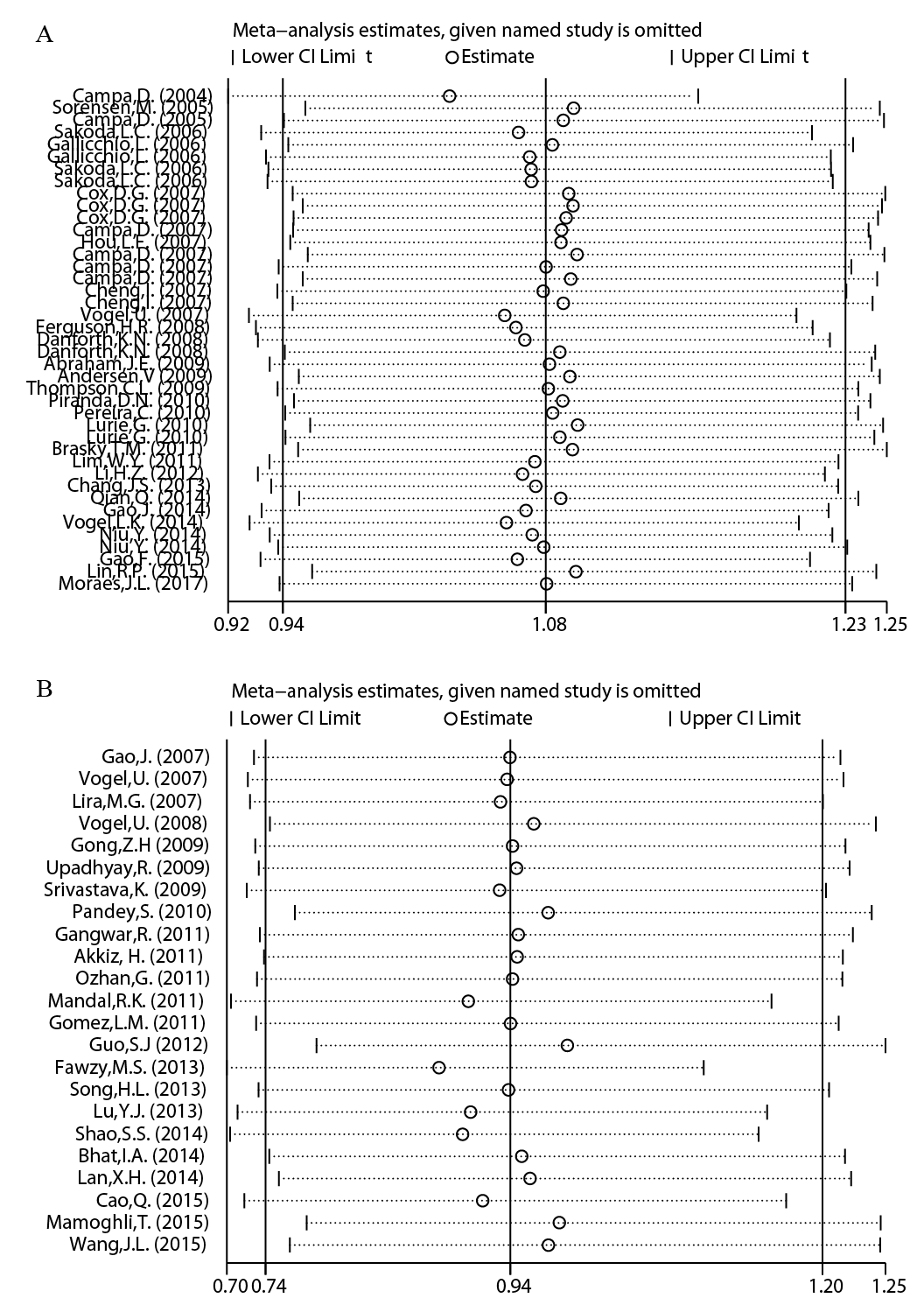

Supplement: Supplementary file 4 — Figure S3. A. Sensitivity analysis of 8473 T > C polymorphism and cancer risk in TaqMan under homozygote comparison. B. Sensitivity analysis of 8473 T > C polymorphism and cancer risk in PCR-RFLP under homozygote comparison. (TIF 4661 kb) [file 12885_2018_4753_MOESM4_ESM.tif]

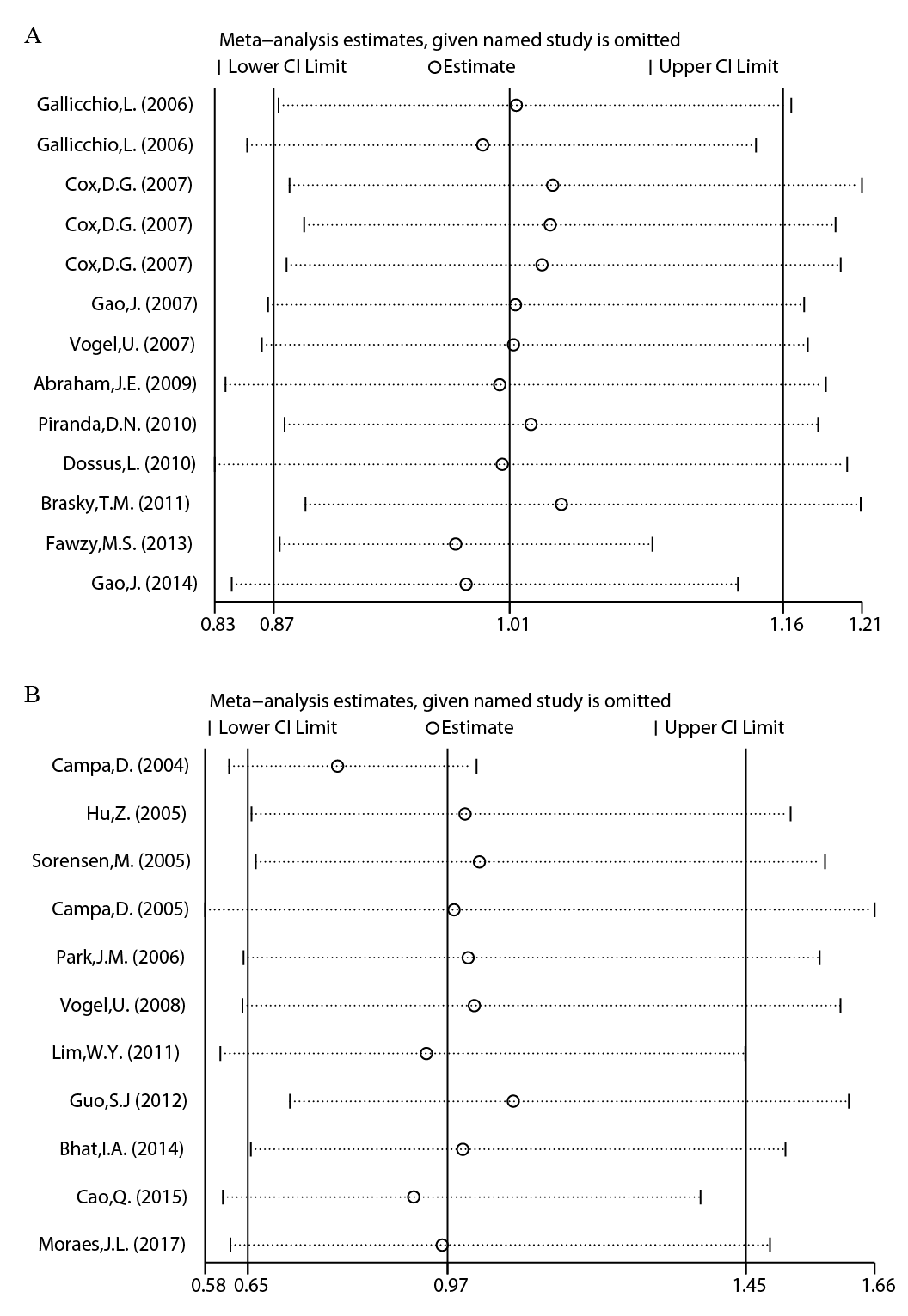

Supplement: Supplementary file 5 — Figure S4. A. Sensitivity analysis of 8473 T > C polymorphism and cancer risk in breast cancer under homozygote comparison. B. Sensitivity analysis of 8473 T > C polymorphism and cancer risk in lung cancer under homozygote comparison. (TIF 4758 kb) [file 12885_2018_4753_MOESM5_ESM.tif]
